# Frequency of deep-seated cerebral microbleeds in patients with lobar hemorrhages and histopathological evidence for cerebral amyloid angiopathy

**DOI:** 10.3389/fneur.2023.1146737

**Published:** 2023-04-12

**Authors:** Monika Huhndorf, Christoph Röcken, Charlotte Flüh, Caroline Weiler, Gregor Kuhlenbäumer, Nora Tegeler, Hannes Schacht, Alexander Neumann, Nils G. Margraf, Ulf Jensen-Kondering

**Affiliations:** ^1^Department of Radiology and Neuroradiology, Universitätsklinikum Schleswig-Holstein (UKSH), Kiel, Germany; ^2^Department of Pathology, Universitätsklinikum Schleswig-Holstein (UKSH), Kiel, Germany; ^3^Department of Neurosurgery, Universitätsklinikum Schleswig-Holstein (UKSH), Kiel, Germany; ^4^Department of Neurology, Universitätsklinikum Schleswig-Holstein (UKSH), Kiel, Germany; ^5^Department of Neuroradiology, Universitätsklinikum Schleswig-Holstein (UKSH), Lübeck, Germany

**Keywords:** CAA, Boston criteria, MRI, histopathology, CT

## Abstract

**Background:**

Cerebral amyloid angiopathy (CAA) is a common disease and the most common cause of lobar hemorrhages in the elderly. Usually, deep-seated microhemorrhages preclude the diagnosis of CAA. In this study, we sought to estimate the frequency of deep-seated microbleeds on MRI in patients with lobar hemorrhages and histopathological evidence for cerebral amyloid angiopathy. In addition, we describe a cohort of patients with cortical and deep-seated microbleeds on MRI and a histopathological specimen available from lobar hematoma evacuation.

**Methods:**

Retrospective database search for histopathological specimens from lobar hematoma evacuation and review of imaging findings (CT and MRI) and patient charts was performed.

**Results:**

Between 1 January 2012 and 31 December 2020, 88 specimens from 88 patients were available. A total of 56 specimens were excluded (no brain tissue in the specimen *n* = 4, other diagnosis *n* = 8, no MRI *n* = 43, and no BOLD-based sequence *n* = 1). Of the remaining 32 patients, 25 patients (78%) did not harbor deep-seated lesions on MRI, of which 17 patients had histopathological features of CAA. A total of seven patients harbored deep-seated CMB. Of these seven patients, three (3/20, 15%) had histopathological features of CAA.

**Conclusion:**

Approximately 15% of patients with histopathologically diagnosed CAA harbor deep-seated microbleeds. This finding may add to the discussion on how to identify patients with CAA and deep-seated CMB.

## Introduction

Cerebral amyloid angiopathy (CAA) is a common disease in the elderly population and the most common cause of lobar hemorrhages in elderly patients (1). Its diagnosis according to the Boston criteria 2.0 requires presentation with cognitive decline, intracranial lobar or subarachnoid hemorrhage, or transient neurological episodes (2). “Probable cerebral amyloid angiopathy,” the category with the highest diagnostic certainty without the need for brain biopsy in the four-tiered system if histological verification is not feasible or desirable, requires the absence of deep-seated hemorrhagic lesions on imaging (CT or MRI).

Strictly deep-seated hemorrhagic lesions are the hallmark of hypertensive arteriopathy (HTN-A). Patients displaying features of CAA and harboring deep-seated CMBs, i.e., mixed location hemorrhages/microbleeds (MLH) (3), are most likely to have a severe form of HTN-A. However, there is growing evidence that CAA and HTN-A may co-exist in a subset of patients. Cortical superficial siderosis (cSS), a strong predictor of CAA, is present in ~ 15% of patients with MLH (4, 5). Moreover, Tsai et al. (6) demonstrated in a cohort of patients with primary intracranial hemorrhages using Pittsburgh compound-B positron emission tomography that patients with MLH and cortical superficial siderosis (cSS) show an amyloid deposition similar to CAA, which further supports possible overlap between MLH and CAA. In the same cohort, deep-seated cerebral microbleeds (CMBs) were present in >50% of patients with lobar hemorrhages. Tsai et al. (7) also reported on the long-term vascular outcome of patients with CAA, HTN-A, and MLH and found MLH in an intermediate position regarding age and ICH recurrence risk. Recently, we could demonstrate that cerebrospinal fluid characteristics in patients with MLH are located in an intermediate position between CAA and healthy controls, i.e., some of these patients may in fact have CAA (8). Therefore, the exclusion of patients with deep-seated CMBs from the diagnosis of CAA may result in some patients being misclassified although the exact number is unknown.

In this study, we sought to estimate the presence of deep-seated CMBs in patients with lobar hemorrhages and histopathology evidence for cerebral amyloid angiopathy.

## Materials and methods

We retrospectively included patients from our radiological, clinical, and histopathological database with the following inclusion criteria: (1) available brain specimen from lobar hematoma evacuation demonstrating either cerebral amyloid angiopathy or no apparent vessel pathology (including other diagnostic work-up such as digital subtraction angiography, MRA, or CTA if available) and (2) available MRI including a BOLD-based sequence within 3 months of the biopsy. Patients with signs of CAA-related inflammation (CAA-ri) were excluded.

**Figure 1:**
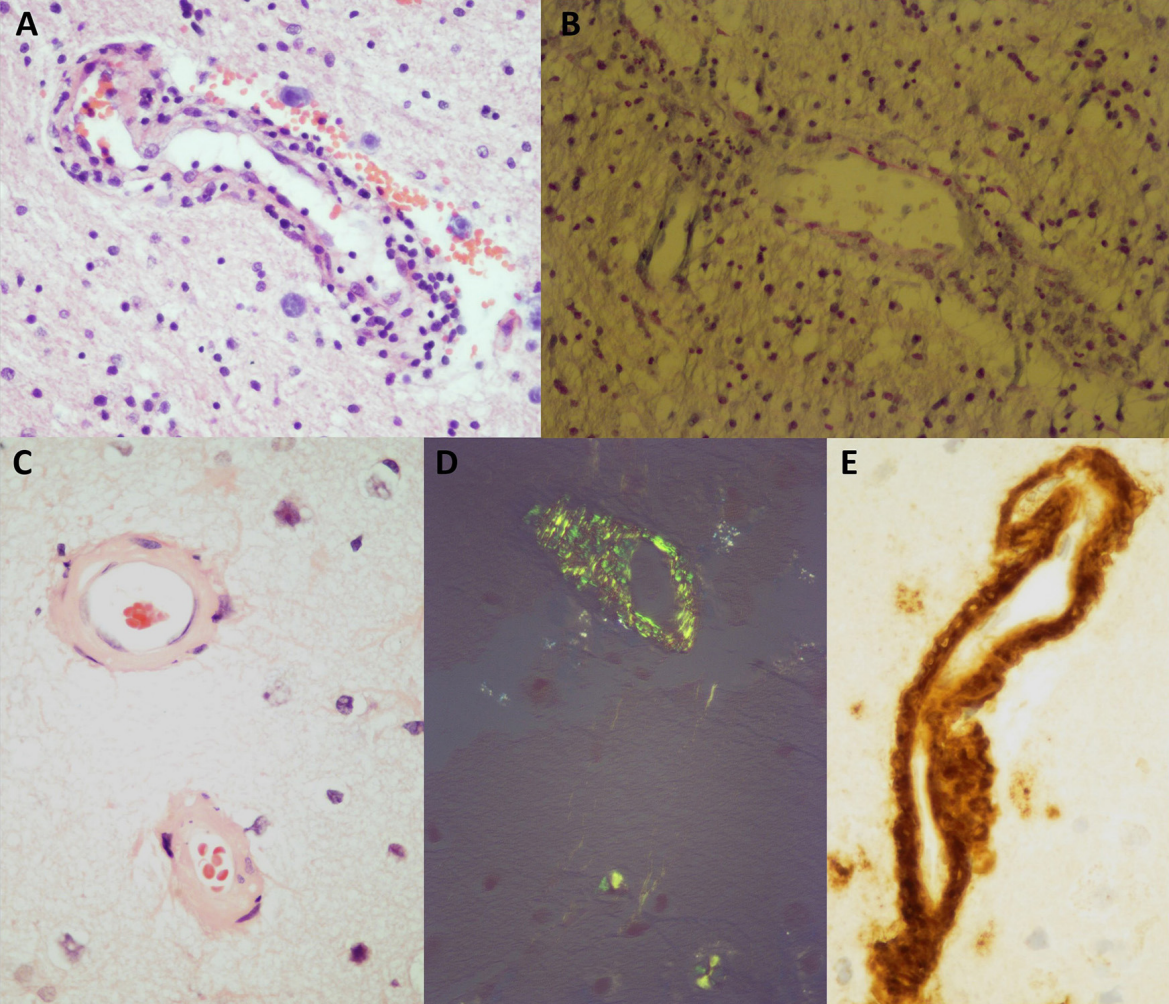
Histopathological findings in CAA and non-CAA specimens. **(A, C)** Hematoxylin- and eosin-stained tissue sections demonstrate vessel wall thickening. **(B, D)** Congo red staining in bright light **(B)** and polarization microscopy **(D)** showing the typical yellow–green birefringence **(D)**. **(E)** Immunostaining against amyloid β demonstrates brown staining in vessel walls.

Hematoma evacuation was performed according to the judgment of the attending neurosurgeon. The obtained brain tissue was processed using standard procedures. The specimens were fixed in formalin and embedded in paraffin stained with hematoxylin and eosin as well as Congo red. If the typical yellow-green birefringence was found using polarization microscopy, additional immunostaining against amyloid β was performed to allow for amyloid subclassification by a board-certified surgical pathologist with a special interest in amyloidosis (CR). Any presence of staining for amyloid β was rated as CAA (9, 10) (Figure 1), i.e., mild, moderate, and severe according to the Vonsattel grading system.

The MLH group was defined by the presence of one or more deep-seated CMB in the brain stem, thalami, or basal ganglia (according to the Microbleed Anatomical Rating Scale (MARS) anatomical template) (11) in which further analysis was undertaken.

**Figure 2:**
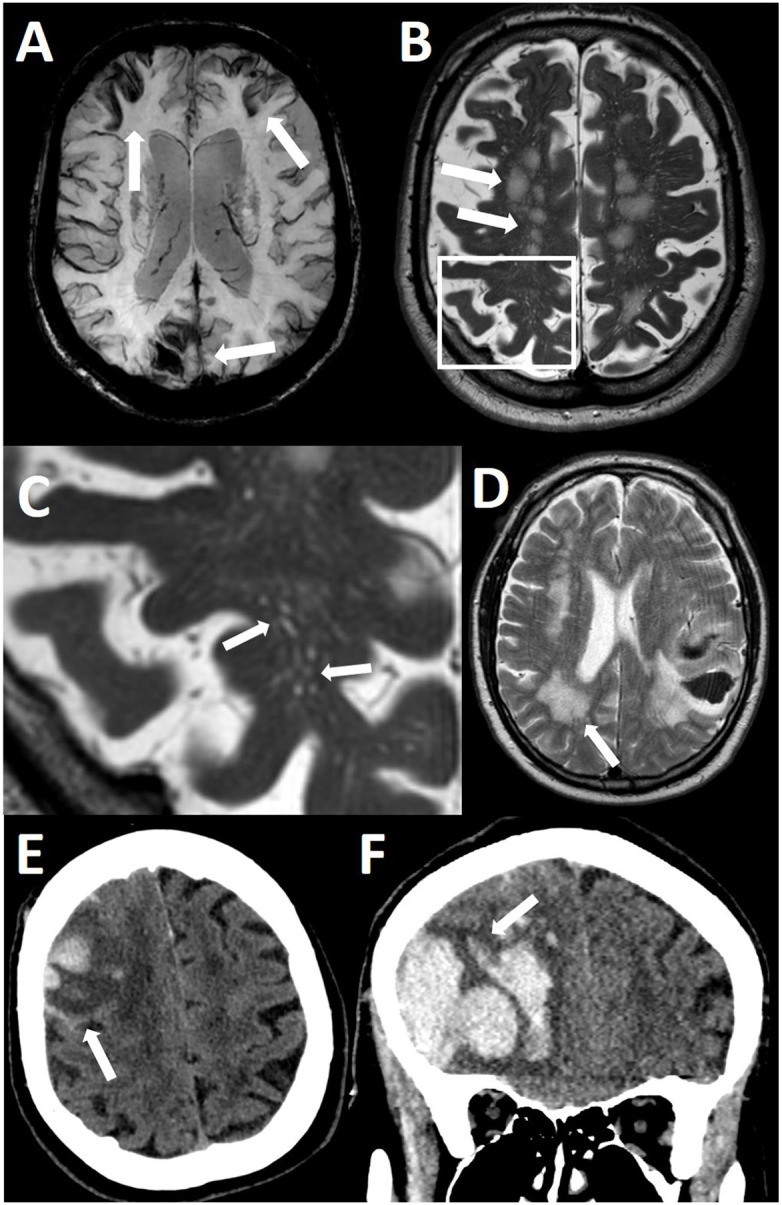
MRI and CT features in cerebral amyloid angiopathy. **(A)** Cortical superficial siderosis (arrows) on an axial susceptibility-weighted image of the brain. **(B)** Subcortical white matter lesions (arrows) on a T2-weighted axial image of the brain. **(C)** Inlet magnification of B, MRI-visible perivascular spaces in the centrum semiovale. **(D)** Dorsal white matter caps (>5 mm, arrow). Note the left parietal lobar hemorrhage. **(E)** Subarachnoid hemorrhage (arrow) adjacent to the main hemorrhage. **(F)** Finger-like projection exiting the main hemorrhage as an extension longer than its width at the exit from the main hemorrhage (arrow).

All MRI scans (including the allocation to the MLH group) were reviewed by a board-certified radiologist (UJK) and a board-certified neurologist (NGM) blinded to history, clinical diagnosis, and laboratory parameters using all available imaging materials in consensus. The presence of the following imaging findings is reported following the STRIVE criteria (if applicable) (12): acute and old infarcts (territorial or lacunar in nature), cSS, and white matter lesions based on the Fazekas score separately for periventricular and deep white matter (13). A total of four mutually not exclusive white matter patterns were distinguished: subcortical, peri-basal ganglia, frontal, or posterior periventricular caps >5 mm (14). Enlarged perivascular spaces were reported in the centrum semiovale and peri-basal ganglia (15). In addition, we performed a manual CMB count separately for lobar and deep CMBs to calculate the ratio number of lobar CMBs/number of deep CMBs (6, 16) (Figures 2A–D).

The CT scans of the acute stage were reviewed by two board-certified neuroradiologists (AN and HS) in consensus regarding the presence of finger-like projections (FLP) and subarachnoid hemorrhage (SAH) adjacent to the acute lobar hemorrhage after completion of the online training using the Edinburgh Criteria (17) for CAA-associated ICH Training (ECCITING) available at https://www.ed.ac.uk/clinical-sciences/edinburgh-imaging/education-teaching/short-courses/training-tools/edinburgh-criteria-for-caa-associated-ich-training. FLP was defined as a hemorrhagic intraparenchymal extension from the main hemorrhage which is longer than its width at the exit point from the main hemorrhage (Figures 2E, F).

The size and location of hemorrhages were measured by one board-certified neuroradiologist (MH) using the ABC/2 or ABC/3 rule for round and irregular hemorrhages, respectively (18).

Demographic and clinical information was extracted from the patient's chart, namely age, sex, history of arterial hypertension, diabetes mellitus, dyslipidemia, antithrombotic drugs and/or statin intake, elevated creatinine, elevated low-density lipoprotein (LDL), and early (within 6 months) ICH-related death.

Variables are displayed using the mean and standard deviation or median and interquartile ranges (IQR) as appropriate. Comparisons were made with a two-sided *t*-test, Wilcoxon–Mann–Whitney *U-*test, chi-square test, or Fisher's exact test as appropriate. The agreement between the two raters for SAH and FLP was calculated using Cohen's κ. A *p*-value of < 0.05 was considered to be statistically significant. R (version 3.5.0, R Foundation for Statistical Computing, Vienna, Austria) was used for all calculations.

The ethics committee of the medical faculty of the University of Kiel approved this retrospective study (B 255/18).

## Results

**Figure 3:**
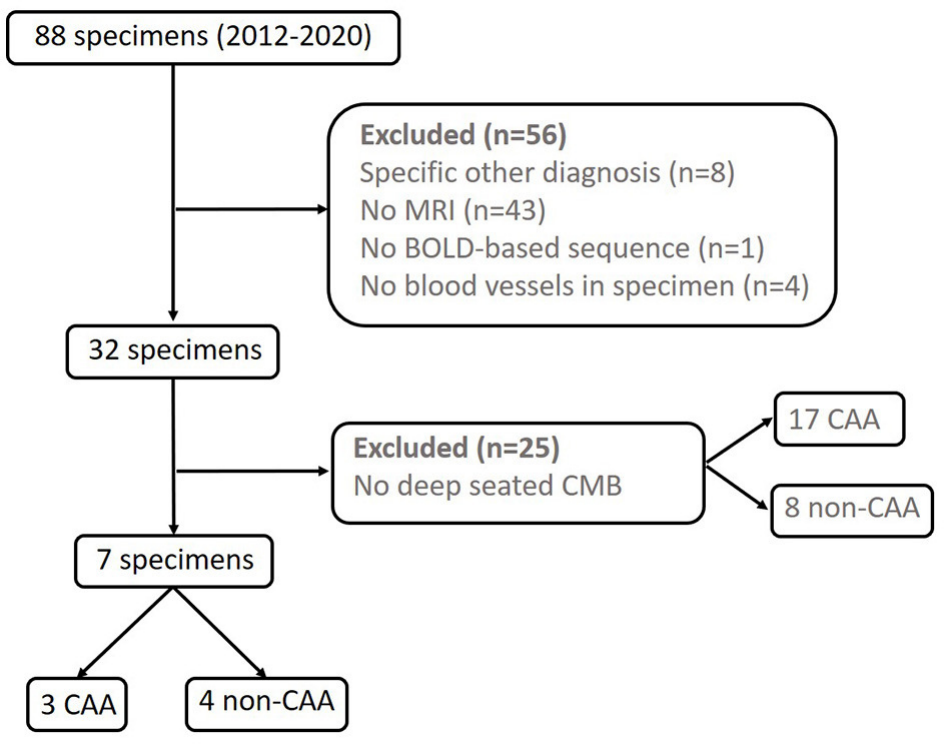
Flowchart of patient selection.

In the period between 2012 and 2021, 88 consecutive specimens from lobar hematoma evacuation were available. In eight patients, a specific diagnosis (hemorrhagic infarct *n* = 3, hemorrhagic tumor *n* = 1, and vascular malformation *n* = 4) was made. In four specimens, no blood vessels were present and were excluded. In 43 patients, no MRI was available. In one patient, an MRI but no BOLD-based sequence was available. Of these patients without MRI, 25 (57%) were diagnosed with CAA. Of the remaining 32 specimens of 32 patients, all patients had an MRI within 3 months of the hematoma evacuation. All MRI scans were performed at 1.5 T including susceptibility-weighted images. A total of 25 patients did not display any deep-seated CMB. Of these 25 patients, 17 were finally diagnosed with CAA (Figure 3).

Of the seven patients harboring deep-seated CMB, three were finally diagnosed with CAA resulting in a prevalence of 15% of deep-seated CMB in patients diagnosed with CAA after hematoma evacuation (3/20 patients). Severity grading was “severe” in all three cases. In four patients, no signs of CAA or any other blood vessel pathology were present. Apart from the presence of deep-seated CMB, all seven patients also had cortical CMBs. Of the seven hemorrhages, five were right-sided: three were parietal, three were frontal, and one was temporal. Typical MRI and CT imaging markers of CAA such as enlarged perivascular spaces, cortical superficial siderosis, and finger-like projections were found both in CAA and non-CAA patients (Table 1). Furthermore, demographics, clinical information, and the detailed results of the rating of the imaging features are shown in Table 1. No patients with CAA-ri were identified in the study.

**Table 1:** Demographics, imaging features, and clinical information.

	**MLH (*n* = 7)**	**Non CAA (*n* = 4)**	**CAA (*n* = 3)**
Age, mean (SD)	69 (8.7)	69 (12.1)	69 (2.8)
Female sex, *n* (%)	2 (29)	1 (25)	1 (33)
**Fazekas score, median (IQR)**			
PVM	3 (2.5, 3)	3 (2.75, 3)	3 (2.5, 3)
DWM	2 (1.5, 2)	2 (1.75, 2)	2 (1.5, 2)
**White matter pattern**, ***n*** **(%)**			
Subcortical	2 (29)	0 (0)	2 (66)
Peri BG	0 (0)	0 (0)	0 (0)
Frontal	1 (14)	1 (25)	0 (0)
Posterior	6 (86)	3 (75)	3 (100)
Lobar lacunes	0 (0)	0 (0)	0 (0)
cSS, *n* (%)	4 (51)	2 (50)	2 (66)
Ratio superficial/deep CMB, median (IQR)	9.33 (2.67, 17)	6.59 (3.26, 9.75)	23 (11.75, 93.75)
**Ischemia**, ***n*** **(%)**			
Chronic	2 (29)	2 (13)	0 (0)
Acute	4 (51)	2 (50)	2 (66)
**EPVS**, ***n*** **(%)**			
BG	1 (14)	1 (25)	0 (0)
CS	2 (29)	1 (25)	1 (33)
FLP, *n* (%)	4 (51)	2 (50)	2 (66)
SAH, *n* (%)	4 (51)	1 (25)	3 (100)
Hemorrhage volume, mean (SD)	70.7 (31.6)	67.0 (41.0)	75.7 (20.2)
Intraventricular extension, *n* (%)	0 (0)	0 (0)	0 (0)
Antithrombotics, n (%)	3 (43)	1 (25)	2 (66)
Statin use, *n* (%)	3 (43)	1 (25)	2 (66)
Early ICH related death, *n* (%)	2 (29)	0 (0)	2 (66)
Elevated creatinine, n (%)	1 (14)	0 (0)	1 (33)
Elevated LDL, *n* (%)	0 (0)	0 (0)	0 (0)
Arterial hypertension, *n* (%)	4 (51)	2 (50)	2 (66)
Diabetes mellitus, *n* (%)	0 (0)	0 (0)	0 (0)
Dyslipidemia, *n* (%)	0 (0)	0 (0)	0 (0)

No statistically significant differences between CAA and non-CAA patients could be detected.

CAA, cerebral amyloid angiopathy, MLH, mixed location hemorrhages/cerebral microbleeds, PVWM, periventricular white matter, DWM, deep white matter, cSS, cortical superficial siderosis, SAH, subarachnoid hemorrhage, IQR, interquartile range, SD, standard deviation, BG, basal ganglia, EPVS, enlarged perivascular spaces, CS, centrum semiovale, CMB, cerebral microbleeds.

Cohen's κ for SAH was 1 (perfect agreement) and 0.72 for FLP (substantial agreement).

## Discussion

In our cohort, 15% of the patients histopathologically diagnosed with CAA according to the Boston criteria 2.0 harbored deep-seated CMB on MRI. However, deep-seated CMB precluded the diagnosis of CAA. If a histological specimen is present, this caveat does not apply, but in clinical practice, a cortical biopsy is only rarely performed. Even in patients with lobar hemorrhages, neurosurgical evacuation is not always warranted.

The prevalence of deep-seated CMB in CAA of 15% in our cohort matches the observation that ~15% of patients with MLH have cSS (4, 5). Other observations also hint toward the fact that a certain percentage may have CAA although the exact proportion is unclear (6–8).

CAA is the consequence of amyloid accumulation in cerebral vessels which renders them fragile and prone to rupture, hence the multitude of possible hemorrhagic manifestations (cerebral microbleeds, intracranial macrohemorrhages, and subarachnoid hemorrhage) (1). For unknown reasons, blood vessels in deep brain structures are relatively spared from these depositions (19). Hemorrhages in deep brain structures are the hallmark of HTN-A which is in the main parts but not exclusively driven by arterial hypertension (20).

Especially in older patients, it is not uncommon and biologically not excluded for CAA and HTN-A to coexist (20). A high percentage of CAA patients is hypertensive (3, 8, 21), and the prevalence of arterial hypertension increases with age. However, this is currently not accounted for in the diagnostic criteria for CAA, the Boston criteria 2.0. In our cohort, two out of four patients with deep-seated CMBs and no histopathological evidence for CAA had cSS and are currently regarded as the single strongest imaging marker for CAA. It is possible that these cases are true false negatives, or in these patients, indeed a dual pathology was present. Other emerging imaging features of CAA were also detected, but the limited number of analyzable patients was too small to obtain clinically reasonable results.

The sensitivity of the original Boston criteria could be increased from 44% (22) to 95% with the modified Boston criteria by incorporating cortical superficial siderosis (23), with a lower sensitivity in the current version (74.5%), incorporating additional white matter markers and a definite description of required clinical presentation. Specificity was very high with the original Boston criteria (100%), which came with a reduction with the modified Boston criteria (81%) and again high specificity with the current version (95%). Furthermore, the sensitivity of the diagnosis criteria is known to depend on the patient cohort of interest. For example, in non-hospital cohorts, the sensitivity of the Boston criteria is known to be lower (24). By ignoring the exclusion criterion of deep-seated CMB, sensitivity could be increased (albeit at an unknown quantity and reduced specificity) and also CT markers in the acute stage of hemorrhage could help (17).

Weaknesses of our study include all the shortcomings associated with the retrospective nature of this study and its limited sample size. As routine medical treatment and diagnostics as per local protocol were performed, a large share of patients had no MRI available which allowed an in-depth analysis with larger case numbers. Furthermore, systematic biopsy or hematoma evacuation was not performed in all patients presenting with lobar hematoma as it was deemed clinically not warranted or not warranted by the local protocol. Moreover, in cases with clear clinical and MRI presentation and even more so in cases with “possible CAA,” a biopsy is rarely performed. In summary, the true prevalence cannot reliably be deducted from the data presented here which can only be regarded as a rough approximation. Although Boston criteria 2.0 do not require immunostaining, it is regarded as the most sensitive technique to detect amyloid deposition. As per the local protocol, routine staining including Congo red and polarization microscopy was performed in all cases. Amyloid-positive cases may have escaped detection but the exact sensitivity of immunostaining in comparison with conventional staining is unknown.

## Conclusion

Deep-seated CMBs are estimated to be present in ~ 15% of patients with CAA. This finding may add to the discussion on how to identify patients with CAA and deep-seated CMB. Future studies should assess the gain and trade-offs in sensitivity and specificity by not excluding patients with deep-seated CMBs.

## Data availability statement

The original contributions presented in the study are included in the article/supplementary material, further inquiries can be directed to the corresponding author.

## Ethics statement

The studies involving human participants were reviewed and approved by Ethics Committee of the Medical Faculty of the University of Kiel. The ethics committee waived the requirement of written informed consent for participation. The participants consented to retrospective analysis of the data collected during routine work-up during the hospital stay. In case the permission could not be retrieved, only anonymized data were used.

## Author contributions

NGM and UJ-K: conception and design. UJ-K, NGM, CR, HS, and AN: formal analysis. UJ-K: wrote the first version of the manuscript. All authors: data acquisition, edited, and approved the manuscript.
